# Exploring needs, barriers, and facilitators for promoting physical activity for children with intellectual developmental disorders: A qualitative focus group study

**DOI:** 10.1177/17446295211064368

**Published:** 2022-02-05

**Authors:** Charlotte Boman, Susanne Bernhardsson

**Affiliations:** Centre for physical activity, Region Västra Götaland, Gothenburg, Sweden; Department of Health and Rehabilitation, Unit of Physiotherapy, Sahlgrenska Academy, Institute of Neuroscience and Physiology, 3570 University of Gothenburg, Gothenburg, Sweden; Department of Health and Rehabilitation, Unit of Physiotherapy, Sahlgrenska Academy, Institute of Neuroscience and Physiology, 3570 University of Gothenburg, Gothenburg, Sweden; Research, Education, Development and Innovation, Primary Health Care, Region Västra Götaland, Gothenburg, Sweden

**Keywords:** attention deficit hyperactivity disorder, autism spectrum disorder, children, intellectual disability, physical activity

## Abstract

**Background:**

Many children with intellectual developmental disorders are insufficiently physically active and do not reach recommendations for physical activity. Pediatric healthcare providers play a key role in addressing these children’s needs, including promoting interventions for physical activity.

**Aim:**

To explore pediatric healthcare providers’ perceived needs, barriers, and facilitators for promoting physical activity for children with intellectual developmental disorders.

**Methods:**

Semi-structured focus groups, analyzed using qualitative content analysis. Sixteen healthcare providers participated.

**Results:**

Main findings are the importance of parental support and engagement, need for structure, and stakeholder collaboration to bridge the gap between pediatric organizations and external stakeholders.

**Conclusion:**

The study highlights the need for developing and implementing strategies to promote physical activity for children with intellectual developmental disorders in pediatric health care, and for producing guidelines regarding physical activity interventions for this vulnerable group.

## Introduction

Insufficient physical activity (PA) is a growing global health concern among children and adolescents (Guthold et al., 2020). Physical activity encompasses all bodily movements that result in increased energy consumption (Caspersen et al., 1985), and occurs in sports and exercise, as well as in everyday activities during school and leisure time, and through active transports. Physical activity has positive effects on physical and mental functions in children, and is also important in relation to risk factors for lifestyle-related diseases that have been detected at increasingly young age, such as overweight/obesity and type 2 diabetes (Berman et al., 2012; Swedish Research Council for Sport Science, 2017). A sedentary lifestyle with insufficient PA is a main contributor to childhood obesity (Wolfenden et al., 2016), a disease that tracks into adulthood (Simmonds et al., 2016; Ward et al., 2017), which underscores the importance of addressing physical inactivity early in life. The WHO recommends that all children between 6 and 17 years should be active, preferably in any aerobic exercise, at least one hour per day at moderate-to-high intensity, and that sedentary time and recreational screen time should be limited (Bull et al., 2020).

Intellectual developmental disorders is an umbrella term for intellectual and neurodevelopmental disorders (Salvador-Carulla et al., 2011). Of these, common disorders are intellectual disability, Autism Spectrum Disorder (ASD), and Attention Deficit Hyperactivity Disorder (ADHD), with a prevalence of 1%, 1.5% and 5%, respectively (Gillberg, 2010). Although the conditions differ in terms of severity and prominence, children with intellectual developmental disorders share common traits such as early motor, behavior and/or cognitive impairments, comorbidity, problems with adjustment to society, and mental ill health later in life (Broberg et al., 2015; Gillberg, 2010).

A considerable and consistent research base shows that PA is beneficial for children with intellectual developmental disorders (Boer et al., 2014; Srinivasan et al., 2014; Stanish and Temple, 2012; Suarez-Manzano et al., 2018). In children with ASD and ADHD, regular PA has an impact on motor, social, behavioral, and cognitive functions (Jeyanthi et al., 2018; Srinivasan et al., 2014; Suarez-Manzano et al., 2018), and in children with intellectual disability, especially on motor, body and mind functions (Boer et al., 2014; Hutzler and Korsensky, 2010; Reza et al., 2013; Stanish and Temple, 2012). However, many children with intellectual developmental disorders are insufficiently physically active and highly sedentary (Hinckson and Curtis, 2013; Khalife et al., 2014; Lobenius-Palmér et al., 2018; Sundahl et al., 2016). Even though aerobic and muscular exercise is recommended for children with intellectual disability, these children, as well as children with ASD and ADHD are less likely to reach PA recommendations (Graf et al., 2004; Khalife et al., 2014; Lauruschkus et al., 2017b; Phillips and Holland, 2011; Professional associations for physical activity (Sweden)., 2017).

Healthcare organizations play an important role in offering adequate lifestyle approaches for individuals with intellectual developmental disorders (Steenbergen et al., 2017). In Sweden, national guidelines recommend that children who are insufficiently physically active should be offered counseling on PA in health care (National Board of Health and Welfare, 2018). Health promotion should be done through counseling, and a person-centered dialog is recommended to promote behavioral change and increase PA and/or decrease sedentary behavior. Working toward sustainable active lifestyle habits already in childhood is invaluable (Biddle et al., 2010; Craigie et al., 2011; Lubans et al., 2011). Because insufficient physical activity and lack of motivation are common in children with intellectual developmental disorders, individualized, structured, and supportive interventions to promote PA would be beneficial (Srinivasan et al., 2014). Health promotion interventions for children and adolescents with intellectual developmental disorders have been sparsely studied, but two small studies have shown promising results of different types of health promoting interventions (Ptomey et al., 2017; An et al., 2019).

When designing and implementing health promotion interventions, needs, barriers and facilitators for the child to participate in PA should be considered. Barriers and facilitators exist at the child, parent, and healthcare provider’s level and include personal, social, environmental, and policy- and program-related factors (Shields et al., 2012). Barriers from the child perspective that make participating in PA challenging include lack of knowledge and physical and social skills related to exercise, activity preferences, lack of friends to participate with, and fear of being stigmatized or attracting unwanted attention. Facilitators of participating in PA include a desire to be fit, healthy, and active, to have fun, practicing skills, and involvement of friends (Shields et al., 2012). For children with intellectual developmental disorders, the condition itself entails specific challenges compared to typically developing peers related to their motor, behavior or cognitive limitations, which may make their participation in certain activities more difficult (Stanish et al. 2016). To understand the children’s strengths and limitations, team-based assessments and interventions are recommended rather than independent interventions from different professions (Gillberg, 2014). The latter is not uncommon, but entails a risk that the child’s complex difficulties are not considered to a sufficient extent (Gillberg, 2014).

Several studies have investigated barriers and facilitators of participating in PA for children with intellectual developmental disorders from the parental perspective. Parental behavior, attitudes to disability, adequacy and proximity of facilities, lack of time, transport, cost, inclusive programs and facilities, and social motivation, have been reported as key barriers or facilitators, depending on the information and education of relevant others, for example, parents and coaches (Columna et al., 2020; McGarty and Melville, 2018; Shields et al., 2012; Sivaratnam et al., 2020). In Swedish children with both physical and intellectual disabilities, the health promotion intervention “physical activity on prescription” (PAP) was investigated and found to be feasible and leading to increased PA levels (Lauruschkus et al., 2017a). Both children’s and parents’ experiences of participating in the intervention were also explored, showing several barriers and facilitators on both individual and environmental levels (Lauruschkus et al., 2015, 2017a).

The perspective of healthcare providers has not been explored to the same extent. For children with physical disabilities, practical limitations, time constraints, and parents’ financial limitations have been reported as important barriers to physical activity participation perceived by healthcare providers, while programs to promote success and inclusion have been reported as important facilitators (Sivaratnam et al., 2020; Wright et al., 2019). For children with intellectual developmental disorders, there is a paucity of studies on the needs and challenges perceived by healthcare providers in working with health promotion interventions. Healthcare providers have been called the missing link to sustainable physical activity participation for children with disabilities, and as having expert knowledge of their young patients’ disability (Brunton, 2017). Elucidating the healthcare provider’s perspective will provide important knowledge that can inform the planning, design and implementation of future health promotion services for this population. Therefore, the aim of this study was to explore pediatric healthcare providers’ perceived needs, barriers, and facilitators for promoting PA in their work with children with intellectual developmental disorders.

## Methods

### Study design and setting

This is an explorative study with a qualitative approach using data from semi-structured focus group discussions. This methodology was chosen because the collective interaction between the participants creates a frame of reference that may lead to better understanding and production of new knowledge of specific topics (Dahlin-Ivanoff and Holmgren, 2017), such as interventions for promoting PA. Because focus groups may highlight the opinions and insights of, for example, employees, the methodology also is appropriate for organizational development (Krueger and Casey, 2009).

The setting for the study was two pediatric organizations in Region Västra Götaland, a Swedish region with approximately 1.7 million inhabitants. The first organization provides specialized care for children in general and the second, habilitation for children with lifelong disabilities; both cater to children 0–18 years old. In and around the largest city in the region, there are 15 pediatric clinics and 11 habilitation clinics (Region Götaland Västra, 2020). The organizations with all their clinics are included in the national and regional healthcare strategy to implement interventions for healthy lifestyle habits for children who are physically inactive (National Board of Health and Welfare, 2018; Nilsson-Green and Manhof, 2018). There is also a regional medical guideline for accountability and collaboration between the organizations, aiming to simplify public healthcare contacts for families (Region Götaland Västra, 2014, 2020). For children who have been issued PAP, specialized PAP clinics are available, offering additional support and coaching.

### Sampling procedure

The two senior managers of the organizations were contacted and five pediatric and four habilitation clinics, all located in urban areas, were selected and included in the study. Both organizations provide healthcare interventions for children with intellectual developmental disorders.

A purposeful sampling strategy was used, aiming to include different professions into heterogenous groups, in order to achieve maximum variation (Dahlin-Ivanoff and Holmgren, 2017). Inclusion criteria were to be a licensed healthcare provider with experience of working with children with intellectual developmental disorders. Invitations to participate were sent to the clinic managers, who distributed it among their staff. Those who fulfilled the inclusion criteria and were willing to participate were contacted by the first author. Each organization had their own focus groups, which ensured homogeneity in the group sessions (Dahlin-Ivanoff and Holmgren, 2017).

### Participants and procedure

Fourteen women and two men, aged between 30 and 58 years, participated in four focus groups (Table 1). The number of healthcare providers from each profession were 4 physiotherapists, 3 occupational therapists, 6 nurses with and without specialist competence in pediatrics, 1 dietician and 2 psychologists. The focus groups took place at the Research and Development center primary health care in Region Västra Götaland, Gothenburg, during working hours, between May and September 2018. The discussions were approximately 1.5 hours per session. The first author, a physiotherapist and Master student with substantial experience of children with intellectual developmental disorders, was the moderator. The second author, a physiotherapist and primary care researcher with experience of qualitative research as well as implementation research, participated as observer. Before the sessions started the participants filled out a form with data on age, gender, profession, and years of experience in the profession (Table 1). The focus groups were conducted using a discussion guide with key questions (Dahlin-Ivanoff and Holmgren, 2017). The key questions, presented below, were formulated in the research group after discussion with professionals in the field and through studying the literature.

**Table 1.** Overview of the participants in focus groups A-D

Focus group	No. of participants	Gender, women/men	Age, range (years)	Years of experience^1^, range	Professions
A	4	3/1	30–55	6–28	2 ROTs, 1 RPT, 1 psychologist
B	3	2/1	33–58	1–25	1 dietician, 1 RN, 1 PT
C	6	6/0	27–50	3–29	2 RNs, 2 RPTs, 1 ROT, 1 psychologist
D	3	3/0	44–45	22–25	3 RNs

^1^Years of experience, i.e years in their profession. ROT= registered occupational therapist, RPT= registered physiotherapist, RN= registered nurse.

Key questions for the focus group discussion:

• *Could you describe how you work to increase PA and decrease sedentary behavior among children with intellectual developmental disorders at your clinic?*
• *What is your experience of different interventions to promote PA for children with intellectual developmental disorders?*
• *What barriers do you perceive when trying to motivate children with intellectual developmental disorders to increase their PA?*
• *What facilitators do you perceive when trying to motivate children with intellectual developmental disorders to increase their PA?*

To introduce the topic, the session started with a “breaking the ice” question (Dahlin-Ivanoff and Holmgren, 2017): “What, in your opinion, characterizes children with intellectual developmental disorders?” The participants were encouraged to discuss openly and relate to the key questions throughout the session (Krueger and Casey, 2009). The observer took field notes and observed the verbal and non-verbal conversation flow. The sessions were recorded digitally and transcribed verbatim by the first author.

### Data analysis

Data were analyzed using inductive qualitative content analysis, according to the classic analysis strategy for focus groups described by Krueger and Casey (2009). The analyzing process started during the focus group sessions, where the authors captured the essence of what was being said. Throughout the process, the aim of the study was continuously kept in mind, directing the course of the analysis (Krueger and Casey, 2009). The analysis was led by the first author and discussed with and verified by the second author in an iterative process, which required several analysis sessions to reach consensus on codes, categories, and themes.

*Step 1* Immediately after each session, the authors reflected together and went through field notes. The first author listened to the recorded discussion. *Step 2* To begin the organization of the data the first author read the transcriptions several times, to familiarize herself with the collective narratives. *Step 3* The first author labeled relevant text with codes that describe the context in accordance with the initial key questions, and the second author verified the coding. *Step 4* The data were then organized into relevant categories. This categorization was done in several sessions in which the codes were compared and contrasted with each other and reorganized many times, until the authors reached agreement on a categorization that was coherent and minimized overlap. *Step 5* A descriptive summary of the categories was written in order to describe what was said. *Step 6* Certain things came up repeatedly and were considered to cut across the questions. They were carried forward into themes, and the report is structured around these themes rather than around the questions (Krueger and Casey, 2009) To illustrate the core of the themes, quotes with essential content were selected, with particular focus on capturing interaction among the participants.

### Ethical considerations

The participants signed an informed consent after receiving written and oral information about the study, including aspects of voluntary participation, the possibility of withdrawing at any time without explaining why, and that the responses to questions would be handled confidentially and presented anonymously. Data are managed and stored according to Region Västra Götaland’s guidelines for storage of research material without public access. Focus group transcripts can be obtained from the corresponding author upon reasonable request. The study was approved by the Regional Ethical Review Board of Gothenburg (Reference number 077-18).

## Results

**Figure 1.**
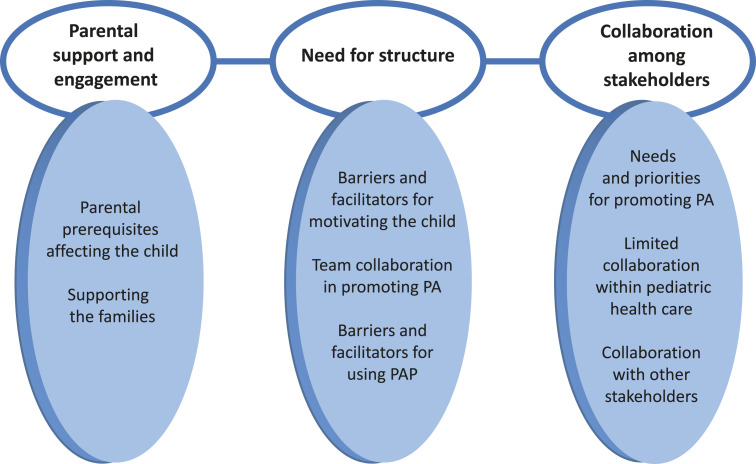
Overview of themes and categories.

The findings were organized into eight categories and three themes that cut across the key questions, summarized in Figure 1. The themes were conceptualized as *Parental support and engagement*, *Need for structure* and *Collaboration among stakeholders*.

### Parental support and engagement

This theme uncovers the supportive needs of children with intellectual developmental disorders and their families. In order to provide adequate care, the participants believed that supporting both parents and children is essential in the promotion of PA.

#### Parental prerequisites affecting the child

A few parental prerequisites were highlighted and described by the participants as central for working with promotion of PA and motivation for children with intellectual developmental disorders and their families. The parental situation and prerequisites, such as prevalence of parental disorders, PA level, self-perceived degree of responsibility, prioritization among the children’s needs, as well as parents’ work situation and financial situation, are all important factors influencing parents’ prerequisites to support a healthy lifestyle in their child.

#### Supporting the families

Individualized parental support from caregivers was described as essential, or else the children are at risk of not getting their needs fulfilled. Medical guidance was also perceived as important to improve the parents’ understanding of their children, for instance, the exclusion of diagnoses like asthma. The participants talked about how, besides investigating symptoms, they considered it important to explain the body’s function and natural reactions to movement, to make the parents more confident in supporting their children to be physically active. To help parents to understand the benefits of PA, the participants perceived a need for providing parental education where these topics could be covered.

It’s so good when the heart beats fast (D2).

Right! (D3).

And when you get all red around the cheeks and sweat in the forehead, that this is the way the body functions (D2).

And this thing when everyone gets out of breath after a run. That is bound to happen (D3).

Yes, exactly! (D2).

Often… What scares them is that they think it’s difficult to breathe but that is the way the body functions when you exert yourself, and that is something to then explain (D3).

Participants also described how they frequently supported parents who also had impairments. They perceived it as far from clear how interventions for promoting PA should be designed to meet the needs of both the children and the parents.

If I use the information with visual support then it’s easier for the parents to get a chance to understand, sometimes the bar is put too high when talking to parents. When I speak to the child the parents also bring up a whole lot that… Just like you said before, there are many parents with similar difficulties (D3). Yeah… (D2). Yes… (D1). Yes, with the visual support it can be very clear and it allows you to be very clear to a child and that favors the parents (D3).

Supporting the family also includes support to the child’s siblings. To stimulate awareness of and practical knowledge about active lifestyle among siblings, the participants suggested group activities for children. Among different age categories, the most difficult category to motivate was perceived to be teenagers.

### Need for structure

In this theme*,* structure, including planning and preparation, is an important prerequisite, or facilitator, for the children to be physically active. The need for structure was apparent in the discussions, both at the individual level of the child, and at the organizational level in which the health promotion intervention is performed in collaboration with parents and healthcare providers. Further, to offer equal pediatric health care, the overall structure of promoting PA needs to be improved and formalized.

#### Barriers and facilitators for motivating the child

The participants perceived barriers for motivation at different levels. At an individual level, the children’s cognitive impairments, ranging from mild to severe, were perceived as a barrier if not approached properly. This was not always easy when not knowing the child well enough, which could sometimes be the case early in the treatment process. Difficulties, such as reduced ability to take initiatives, sedentary behavior, and excessive screen use, were often experienced in these children. The participants also described common occurrences of inability to understand the concept of health and sustainable lifestyle behavior. At the organizational level, healthcare providers’ awareness of the child’s strengths and limitations was perceived as an important facilitator, to compensate the children’s earlier negative experiences of PA. At a societal level, unfamiliar environments were regarded as barriers.

It might be more difficult to focus in a conversation, [the child] seldom sits still for long to enable the conversation… (C3).

I think they might need a little more visual support, for it to be concrete, as you said, it’s hard to understand (C2).

It demands a lot more of me in preparation and that I know the child a little since before (C5).

I also think of what you mentioned before that it’s difficult to think about consequences also when you have an intellectual disability,… even more for them than for someone else in that age, you know ”I will do this to reach this,” or ”in some years I will feel so much better if I do this” (C3).

Yes exactly. //…// It’s the same thought maybe with other activities or with PA (C2).

The participants considered cognitive support as the most important facilitator for increasing motivation. Through cognitive support they perceived they were able to communicate a clearer picture of a proposed activity and of what was expected from the child, allowing a better understanding of why the activity could be fun, meaningful, and fit a specific child. They described their work with visual material, such as pictures or objects, as well as concretizing, structuring, and preparing before an activity. To awaken the child’s internal motivation many used motivational interviewing (MI). Other facilitators, such as PAP, a support person, adapted activities, and fast rewarding screen-related activities, were also mentioned.

#### Team collaboration in promoting PA

Participants perceived team collaboration as a prerequisite for promoting PA for children with intellectual developmental disorders. Collaboration occurs at team meetings and together with the children and parents. The participants described several types of interdisciplinary collaboration for promoting PA, classified as specific or general. Specific interventions are cognitive support, PAP, MI, and information about the effects of PA. General interventions are onward referrals to activities within or outside pediatric health care. The occupational therapists’ main contribution was described as providing prerequisites such as cognitive support, structure, and routines. For nurses it is MI, PAP, and onward referrals. Physiotherapists work with PAP, motivation, information, and onward referrals. The psychologists provide parental support, MI, behavioral activation, and onward referral. Dieticians usually pass inquiries about PA on to other professionals.

I think if we are to work with activities during the week I might be able to help to find that first way of creating an overview over all the activities in the week, perhaps a visual schedule. //…// balance during the day (C2).

I guess, it’s where we nurses also come in, in these basic things when we talk about sleep, for example, and this thing if they move they might get better sleep, if they get a bit tired physically. //…// I guess, it’s the way we enter the starting of a conversation about PA (C4).

For us physiotherapists, it’s much about referring onward to any good sport that we talked about (C3).

Yes, I feel that’s the way to do it somehow… or find ways to make the activity pleasurable (C5).

We often work together with other professions but for me the duty is then often about counseling, //…// I get to see the parents and try to, in different ways, to find the positive things that the child fancies and also ways to enter leisure time activities (A2).

The participants meant they normally inform about general health effects of PA, especially for mental wellbeing, but not specifically for children with intellectual developmental disorders. They perceived a need to increase their knowledge about PA for these children.

#### Barriers and facilitators for using PAP

The consultative intervention PAP came up consistently in the discussions as an intervention commonly used to increase physical activity among the children. Although PAP is not a formally implemented intervention in either of the two participating organizations, the participants described that it is used to various extents, depending on personal preferences and competencies. Most participants expressed positive attitudes toward PAP, but also stated that they had limited experience of using the intervention. Healthcare providers who use PAP were perceived by their colleagues as enthusiasts, with a devotion to physical activity, and were found among any profession, for example, physicians, nurses, or physiotherapists. Their use of PAP could pave the way and facilitate for others to use the intervention.

The most common way to use PAP is to recommend PA and issue a written prescription for an activity, and then refer the families onward to external stakeholders at sport centers or leisure time activity organizers. Another strategy described is to have a person-centered dialog with the child and their family, before issuing a written prescription. However, the lack of time and resources to have this person-centered dialog, as well as proper follow-ups with the families, was frequently discussed as an important barrier.

One of the children who’s been coming to me, is in [her/his] teens with intellectual disability where… there is a really functioning parental engagement. Then we exercised and then at the same time she had contact with the nurse because of overweight and the nurse made a written prescription of PA so now they bought a membership in a gym. And I have been there once to show them exercises. First, they went on their own and then I came. Then I will go there again in a while and this feels like a spot-on example (A4).

Right (A1).

Yes (A2).

Other barriers for using PAP were perceived uncertainties regarding its formal structure, routines and responsibilities, and clinical pathways in pediatric healthcare.

Who issues PAP, is it the physiotherapist? (A2).

Yes. And nurses, doctors and I don’t know… And occupational therapists might be able to do it, that I don’t know (A1).

Further, the participants expressed concern related to the transition between health care and external contexts, where they perceived a lack of coordination with the external stakeholders that organize activities for the children.

### Collaboration among stakeholders

### Needs and priorities for promoting PA

The participants all agreed on the importance of working with promotion of PA for children with intellectual developmental disorders in pediatric health care. Although the topic, and especially PAP, was regarded as “young,” it was considered to cater to the needs of the children as well as to the parents’ requests and expectations. To raise the effectiveness of PAP and other interventions promoting PA, the participants perceived they need to be more formalized than what they are today. The importance of collaboration with physiotherapists was mentioned, especially for children with intellectual developmental disorders who need considerable support to become physically active. Some described close team collaboration and others referred onward to external physiotherapists working in the vicinity where local leisure time activities were arranged. Local and accessible activities were perceived as a facilitator for the families when engaging their children in PA, but the procedures for onward referrals were perceived as unclear or generally not well known.

We can prescribe PA and encourage parents to activities for their children. But we as an organization, we don´t provide much. We have no physiotherapists connected to us (D1).

No (D2).

We have been using… //…// we have had a lot to do with the PAP clinic. We’ve got an immense amount of support from those physiotherapists working in the vicinity (D1).

To be close is of great importance… (D2).

Yes… (D1).

#### Limited collaboration within pediatric health care

The participants experienced that there is only sporadic collaboration regarding PA and children with intellectual developmental disorders among the different organizations within pediatric health care. The little collaboration there is, is not primarily about lifestyle habits or promoting PA but rather about medical issues. The participants described two forms of collaboration that could involve promoting PA; one aiming to get an overview of complex children and the other being an agreement for children with overweight or obesity. The participants pointed out that the collaboration among the pediatric organizations could be better.

There are so many children who don’t get remitted //…// but who has the right to come, I think. It has such a low priority “yes, yes but there is so much else, God, do we have to think about that also?” Yes, but this teenager has very high blood pressure. It could be the obesity, might not be the medicine, it might be the obesity because this teenager weighs 264 pounds. It has such a low priority so I guess there’s no time for such a collaboration (C4).

But that is a good collaboration isn’t it, that you help remitting then they can take over (C6).

To avoid that children and their families get juggled around and to enable good collaboration for promotion of PA, the participants suggested better structure in pediatric health care. This could be, for example, in the form of guidelines for working with lifestyle habits and a general increase of knowledge about the effects of PA for these children.

It takes time and money, resources to be able to profile oneself and an organization in the right way so that others can understand what you’re good at. And it’s in one way, as you mentioned before, it needs to come from above also (B1).

To build a solid structure so that you know where to turn … (B2).

Yes, it would interlink a little better yes, uh-huh… (B3).

So that no one falls between the chairs… (B2).

#### Collaboration with other stakeholders

A huge challenge perceived by the participants is to bridge the gap between pediatric health care and other stakeholders, such as school healthcare clinics, primary care rehabilitation units, specialized PAP clinics, and leisure time activities. They meant that parents need to take on much of the responsibility to bridge this gap, which is not always easy. When the parental support is insufficient, the participants described that it was difficult to collaborate with other stakeholders. They perceived a need for interventions that is not fulfilled by any profession today. Even though it is not in their regular duties, physiotherapists sometimes fill the gap to support the families.

One big problem I think //…// it becomes a huge job for the parents (C1).

Many might have tested some time to take part in a regular exercise group or club or so, but it hasn’t worked. And then you still try, or at least I as a physiotherapist do, I try to recommend others that I know have customized groups. But then I get into this situation where I might not have the possibility to join in and refer onward. It gets stuck along the way where… they get tasks, they get possibilities to take contact and then nothing happens because life gets kind of in between. (C5).

Other collaborating stakeholders that were mentioned are Social Service and agents operating according to the law of “Support and service to certain people with disabilities,” for families in need of extra support. The outcome of these interventions aiming to promote PA were perceived as much varied, depending on the prerequisites within the family.

## Discussion

The main findings of this focus group study of sixteen healthcare providers suggest that support and engagement from parents, structure, and collaboration among stakeholders play important roles as facilitators for the promotion of PA for children with intellectual developmental disorders. The family situation is perceived as critical for motivation, and team contributions necessary to promote an active lifestyle. Stakeholders need to collaborate both internally among different pediatric healthcare units and externally with other organizations. The study shows that interventions to promote PA for children with intellectual developmental disorders cannot be understood from any single perspective, but need to be viewed in a wider context. From an ecological perspective, micro, meso and macro levels that are individual, organizational, and societal, all constitute contextual factors that influence the healthy development of a child (Bronfenbrenner, 1975). These levels could also be traced in the themes of this study.

The participants viewed *parental support and engagement* as an important facilitator, crucial for promotion of PA. The need for parental support is consistent with earlier research from the parental and child perspective, in children with and without intellectual developmental disorders (Broberg et al., 2014; Columna et al., 2020; Edwardson and Gorely, 2010; McGarty and Melville, 2018; Steinhart et al., 2021). An example of parental support that has been proposed in earlier research is parent education related to the benefits of physical activity and the importance of providing opportunities for the child to be active (McGarty and Melville, 2018). In their qualitative study exploring both the child, parent, and professional perspective, Steinhart et al (2021) found parental support to be the most important facilitator for participation in PA and other leisure activities. Hutzler and Korsensky (2010) have suggested that family support, but also a wider range of social support, for example, through peer modeling, is essential to achieve a behavior change toward an active lifestyle, because of the great risk of falling back into previous patterns. The participants in our study expressed that many of the children’s parents also show signs of impairments, indicating the importance of supporting not only the child but also the parents when working with PA promotion. Cognitive difficulties and ASD are overrepresented among parents of disabled children (Broberg et al., 2014) and depression and anxiety have been associated with parenting children with intellectual developmental disorders (Scherer et al., 2019), potentially affecting the children’s possibilities to be physically active and further stressing the need for cognitive parental support from healthcare professionals.

Teenagers were perceived by the participants in our study as the most difficult age group to motivate. Adolescence is a trying period for many families and many teenagers have developed overweight/obesity and a sedentary behavior (Broberg et al., 2014; Emerson, 2009; Erhart et al., 2012; Höckertin et al., 2016; Srinivasan et al., 2014). In adolescence, healthcare contacts normally decrease for disabled children (Broberg et al., 2014), reducing the availability of health promotion interventions.

Our findings suggest that parental support in promotion of PA for children with intellectual developmental disorders should be educative, individually adapted, and sustained throughout childhood and adolescence. The importance of individual adaptation has been described also from the parental perspective (Alesi and Pepi, 2017; Columna et al., 2020). Columna et al. (2020) showed in their review that parents of youth with disabilities want closer collaboration and communication with professionals to increase their knowledge about PA and to be better able to support their children in being physically active. Lauruschkus et al. (2017a) described similar findings in their study on parents of children with cerebral palsy who had participated in a PAP intervention.

At the individual level, the children’s cognitive impairments were considered a barrier for being physically active, implying a *need for structure* of the child’s day and thorough preparation of their activities, as well as cognitive support for motivating the child. In their systematic review of barriers and facilitators for physical activity among children with physical or intellectual disability, Shields et al. (2012) also identified barriers related to the child’s impairment, particularly negative attitudes to disability. Sensory impairments were identified by Sivaratnam et al. (2020) as a barrier to participation in PA perceived by parents of children with cerebral palsy and ASD. In the review by Columna et al. (2020), characteristics of the disability were a frequent barrier to PA described by the parents of children with physical disabilities. These findings highlight the need for tailoring activities to suit the broad range of abilities in children with cognitive, sensory, or physical impairments.

To address the need for structure, as well as the aspect of behavioral change, an adapted version of MI that includes cognitive support could be a way to increase motivation for the families, as has also been suggested by Oritz and Sjölund (2015). To better understand the children’s complex needs, team collaboration is highly preferred when promoting PA, and also recommended for children with intellectual developmental disorders where different professions can contribute with their specific competence (Gillberg, 2014).

At the organizational level, the participants in this study described a need for better structure when promoting PA. The need for structure has also been identified by clinicians working with children with cerebral palsy, who highlighted the need for a centralized system of communication for parents and professionals involved in the child’s care (Sivaratnam et al., 2020). Even though the organizations are targeted in Region Västra Götaland’s healthcare strategies for the promotion of healthy lifestyle habits for physically inactive children, including children with intellectual developmental disorders, this work still seems to be in its infancy at both an organizational and societal level. The urban clinics included in this study already benefit from having an established support for counseling physical activity through a specialized PAP clinic, as well as a more developed catalog of local activities and sports arenas than the other clinics.

*Collaboration among stakeholders* was considered a crucial facilitator, but a challenging task when it comes to interventions to promote PA. This challenge was identified also by the clinicians in the study by Sivaratnam et al. (2020), who expressed a need for better communication among parents, professionals and coaches at sports clubs involved in the child’s care and activities. In Sweden, there is no consensus regarding how the healthcare system can meet the complex needs of children with intellectual developmental disorders (Broberg et al., 2015) or where in the healthcare system they should be managed and how organizations should collaborate.

At the organizational level, physiotherapists were perceived as a key profession in the work with promotion of PA in our study, but they are rarely involved in pediatric health care. Instead, they are often found in other healthcare organizations such as primary health care, which was considered problematic by the participants. Earlier research has shown that traditionally, the physiotherapist’s role in the treatment of children with intellectual developmental disorders is limited, compared with other children needing pediatric health care (Lauruschkus et al., 2017a). Because physiotherapists’ skill set includes bodily aspects and adapted physical exercise (Wikstrom-Grotell and Eriksson, 2012), their competence in teams for children with intellectual developmental disorders seems warranted.

At the societal level, the participants perceived a gap between pediatric health care and external stakeholders in different arenas. The risk of “falling between the chairs” was perceived as high for children with intellectual developmental disorders in need of support for an active lifestyle. Public health care catering to the needs of disabled children and their families’ needs to collaborate in supporting parents to organize their children’s activities, thereby reducing the parental burden (Broberg et al., 2014), in line with the findings of this study. To enable interventions promoting PA across organizational boundaries, strengthened collaboration among stakeholders is needed. This finding is supported by Shields and Synnot (2016) and underscores the need for clear management pathways among different stakeholders. At the environmental level, our finding that easy access to activities is an important facilitator, is supported by previous research that has identified both proximity of location and accessible facilities as facilitating PA among children with physical disabilities (Lauruschkus et al., 2015; Shields et al., 2012).

The themes can be mapped to the domains of the Normalization Process Theory (NPT) (May et al., 2009), which is helpful in interpreting the study findings and understanding which domains that need to be addressed when designing a PA intervention and how it can be routinely embedded (normalized) in healthcare practice (McEvoy et al., 2014; May et al., 2018). The NPT posits that the embedding process is structured around four domains: coherence (participants’ understanding of the process), cognitive participation (commitment to working with the intervention), collective action (efforts to use the intervention), and reflexive monitoring (evaluation of the intervention). The theme *Parental support and engagement* could be seen as reflecting collective action, where healthcare providers promote PA using different counseling strategies, such as PAP, to support the families to support their child in becoming more active. The theme *Need for structure* could fit into the domains coherence and cognitive participation, which aim to raise awareness of the benefits of promoting PA and of its contextual integration in pediatric health care. The *Collaboration* theme emphasizes collaboration between healthcare providers and families, but also the integral relation between staff and managers reflecting the domain collective action (May et al., 2007; May et al., 2018). The last NPT domain, reflexive monitoring, was less applicable because promotion of PA is not yet embedded in the organizations and evaluation is not systematized. Some of our themes could fit into several domains, which is a known challenge in using the NPT as several of its domains seem to overlap (May et al., 2018). For example, the themes *Need for structure* and *Collaboration between stakeholders* may pertain to the domains of collective action and cognitive participation.

PAP was perceived by most participants as a structured intervention to support the children and their families to increase their PA levels. However, the intervention has not been studied in this population and is therefore difficult to implement on a larger scale. PAP has been investigated in children with cerebral palsy, of which many also had intellectual disability, and shown to be feasible (Lauruschkus et al., 2017a), but would need to be studied further before any systematic implementation. The findings of our study indicate that efforts promoting PA need to be structured and integrated in team-based interventions.

Our findings indicate a need for guidelines regarding interventions promoting PA for children with intellectual developmental disorders in pediatric health care. Guidelines could be developed through structured interdisciplinary collaboration among stakeholders using healthcare-adapted implementation strategies focusing on parental support. For improved structure and dialog among stakeholders, an interdisciplinary, inter-agency approach could be a way forward. Further research on, for instance, effective strategies to promote PA in this vulnerable group of children is warranted and could draw on the findings of this study. There is also a need to explore the organizational perspective of unit and senior managers of healthcare organizations on promotion of PA in pediatric health care.

The focus group method was chosen to allow different professions to reflect collectively upon the topic and to bring up the social context influencing their work. Focus groups can be used to gather important knowledge to guide decision making before an intervention (Krueger and Casey, 2009). Physical activity was not an unfamiliar topic to the participants, but they had not previously discussed it together. Since the focus group method is based on shared experience, it is important to put together homogenous groups (Krueger and Casey, 2009). This was accomplished by conducting separate sessions for the two organizations, which aimed to sample collective views from participants from the same organization and also to avoid comparisons between the organizations. Homogeneity was further achieved through the participants’ common experiences of working with the target group and through their working in the same organization with its common management, organizational culture, and mission. Heterogeneity was achieved through the range of professions with variations in age, sex, and year in their profession (Dahlin-Ivanoff and Holmgren, 2017). In one group there were three participants from the same profession, which potentially could be a limitation since they only represented one perspective. However, this was also a strength because it enabled a valuable discussion, which provided meaningful input to the data collection. The aim of the study was to get a general picture of the views of different healthcare professionals, but it is also valuable to gather participants from the same profession, to reach a further depth in the discussions (Dahlin-Ivanoff and Holmgren, 2017).

Another study limitation is that no physician participated. Physicians also promote PA, and it would have been valuable to also get their views. Because promotion of PA is not considered formalized enough, it was challenging to recruit enough participants for the focus groups. The number of participants could be seen as small, but smaller groups with three to six participants have been shown to be very dynamic; engagement in the discussions is more important than the number of participants (Krueger and Casey, 2009).

Because of the qualitative design used in the study, the findings cannot be generalized widely. However, the use of systematic procedures to collect and analyze data strengthens the trustworthiness of the study, and may enable the findings to be transferred to other, similar, contexts (Krueger and Casey, 2009). Transferability is also enhanced by the presentation of relevant categories, themes and by quotes taken directly from the discussions. Credibility and dependability were strived for through detailed descriptions of the analytical process and recurrent summations during and after the discussions.

## Conclusions

This study contributes new knowledge on perceptions of promotion of PA in children with intellectual developmental disabilities among a sample of pediatric healthcare providers in Sweden. The findings indicate the importance of parental support and engagement and structured team-based interventions to meet the needs of the children and their families. The findings also indicate a need for structure at an organizational level where promotion of PA could be further developed. At a societal level there is a need for investigation of effective ways to promote PA in this vulnerable group of young individuals, and for a more structured collaboration in the transition between pediatric health care and external stakeholders in order to provide adequate care pathways. The study highlights the need for, and can provide the basis for, the development and implementation of strategies to promote PA for children with intellectual developmental disabilities.
